# ASO Author Reflections: Robotic Proximal Gastrectomy with Double-Tract Reconstruction and En-Bloc Distal Pancreatectomy: Balancing Oncologic Efficacy and Functional Preservation in Locally Advanced Gastroesophageal Junction Tumors

**DOI:** 10.1245/s10434-026-20004-9

**Published:** 2026-06-18

**Authors:** Anneliese N. Hierl, Naruhiko Ikoma

**Affiliations:** Department of Surgical Oncology, The University of Texas MD Anderson Cancer Center, Houston, TX USA

## Past

Surgical management of locally advanced gastroesophageal junction (GEJ) tumors demands careful oncologic planning, particularly when adjacent organ invasion necessitates multivisceral resection. Total gastrectomy has long served as the default operation; however, its associated nutritional deficiencies, hormonal consequences, and impact on quality of life have prompted interest in stomach-preserving alternatives.^1^ Proximal gastrectomy offers remnant gastric preservation but has historically been limited by problematic reflux and anastomotic complications when reconstructed with a direct esophagogastric anastomosis.^2^ Double-tract reconstruction (DTR) addresses these concerns by routing enteric flow through both the gastric remnant and a jejunal limb, with recent trials and meta-analysis confirming lower reflux and stricture rates with survival equivalent to total gastrectomy.^3,4^ When pancreatic invasion is present, the added complexity of en-bloc distal pancreatectomy further raises the technical stakes. Whether function-preserving reconstruction could be safely combined with robotic multivisceral resection in this setting remained unknown.

## Present

In our recently published video,^5^ we demonstrate robotic proximal gastrectomy with DTR and en-bloc distal pancreatectomy with splenectomy in a 70-year-old male with a Siewert Type II GEJ adenocarcinoma and direct pancreatic invasion. Our surgical approach is guided by several key principles:

1. careful patient selection following preoperative chemotherapy response assessment, ensuring appropriate candidacy for function-preserving resection;
2. meticulous mediastinal and oncologic lymph node dissection to achieve R0 resection without compromising oncologic efficacy;
3. hand-sewn esophagojejunostomy with appropriate spacing between the esophagojejunostomy and gastrojejunostomy to optimize functional outcomes and reduce anastomotic complications; and
4. upright fixation of the remnant stomach to facilitate emptying of food into the jejunum and systematic closure of hiatal, mesenteric, and Petersen defects to prevent internal herniation.

The robotic platform facilitated precise dissection in both the mediastinum and upper abdomen, and the procedure was completed with R0 resection achieved. The patient had prompt postoperative recovery, supporting early resumption of systemic therapy, which is an important consideration given his advanced disease.

## Future

Given the rarity and heterogeneity of GEJ tumors with pancreatic invasion, large prospective comparative studies are unlikely to be feasible. Future efforts should focus on collaborative multicenter experiences to better define patient selection, technical considerations, perioperative safety, and oncologic outcomes of function-preserving approaches in selected patients. Incorporation of patient-reported and nutritional outcomes may provide important complementary measures beyond traditional morbidity and survival endpoints. In addition, lessons from broader studies of GEJ cancer surgery without pancreatectomy may help optimize reconstruction strategies and perioperative management for this rare subgroup requiring multivisceral resection.

Ultimately, for carefully selected patients with locally advanced GEJ tumors, robotic proximal gastrectomy with DTR and en-bloc distal pancreatectomy and splenectomy, resection may offer an approach that achieves oncologic efficacy while preserving physiologic function and supporting timely return to systemic therapy.
